# Advances in Detecting Ciguatoxins in Fish

**DOI:** 10.3390/toxins12080494

**Published:** 2020-07-31

**Authors:** Tibor Pasinszki, Jimaima Lako, Todd E. Dennis

**Affiliations:** 1 Department of Chemistry, School of Pure Sciences, College of Engineering, Science and Technology, Fiji National University, P.O. Box 3722 Samabula, Suva, Fiji; 2 Department of Food Technology and Home Economics, School of Applied Sciences, College of Engineering, Science and Technology, Fiji National University, P.O. Box 3722 Samabula, Suva, Fiji; jimaima.lako@fnu.ac.fj; 3 Department of Biology, School of Pure Sciences, College of Engineering, Science and Technology, Fiji National University, P.O. Box 5529 Lautoka, Fiji; todd.dennis@fnu.ac.fj

**Keywords:** ciguatera, ciguatoxin, cytotoxicity assay, ELISA, HPLC, immunoassay, LC-MS/MS, mouse bioassay, receptor-binding assay

## Abstract

Ciguatera fish poisoning (CFP) is currently the most common marine biotoxin food poisoning worldwide, associated with human consumption of circumtropical fish and marine invertebrates that are contaminated with ciguatoxins. Ciguatoxins are very potent sodium-channel activator neurotoxins, that pose risks to human health at very low concentrations (>0.01 ng per g of fish flesh in the case of the most potent Pacific ciguatoxin). Symptoms of CFP are nonspecific and intoxication in humans is often misdiagnosed. Presently, there is no medically approved treatment of ciguatera. Therefore, to mitigate the risks of CFP, reliable detection of ciguatoxins prior to consumption of fish tissue is acutely needed, which requires application of highly sensitive and quantitative analytical tests. During the last century a number of methods have been developed to identify and quantify the concentration of ciguatoxins, including in vivo animal assays, cell-based assays, receptor binding assays, antibody-based immunoassays, electrochemical methods, and analytical techniques based on coupling of liquid chromatography with mass spectrometry. Development of these methods, their various advantages and limitations, as well as future challenges are discussed in this review.

## 1. Introduction

Ciguatera fish poisoning (CFP), currently the most common marine biotoxin food poisoning worldwide, is a non-bacterial foodborne disease associated with consumption of circumtropical fish and marine invertebrates that are contaminated with polyether sodium channel activator neurotoxins (ciguatoxins, CTXs) [1,2,3,4,5,6,7,8,9,10,11,12]. CTXs are a family of heat-stable and lipid-soluble compounds that cannot be degraded by normal cooking. CTXs are colorless and odorless, therefore cannot be detected by smell or visual inspection of fish flesh. Ciguatoxins are produced by certain benthic dinoflagellate species from the *Gambierdiscus* and *Fukuyoa* genera and enter the marine food chain via herbivorous fish and invertebrates [2,5,9,13]. These toxins are subsequently biotransformed in herbivorous, omnivorous, and carnivorous fishes to more oxidized and more potent forms of CTXs and accumulate to toxic levels in edible fish. During the biotransformation of P-CTX-4B to P-CTX-1 (see below) there is a ten-fold increase in potency [8]. The structure of CTXs varies according to geographic distribution; therefore, they are classified as Pacific Ocean (P-CTX), Caribbean Sea (C-CTX) and Indian Ocean (I-CTX) ciguatoxins. P-CTX-1 is regarded as the most potent toxin, and the recommended safety limit for CTXs in fish for human consumption has been set at 0.01 ng P-CTX-1 toxin equivalent/g fish tissue (0.01 ppb P-CTX-1 equivalent) by both the European Food Safety Authority (EFSA) and United States Food and Drug Administration (US FDA) [1,14]. The recommended safety level for C-CTX-1 equivalent toxicity is 0.10 ppb [1,9,14]. Although the safety limit for I-CTXs has not been published yet, based on experiments indicating that the toxicity of I-CTX-1 is 60% of that of P-CTX-1 potency [15], a safety level of 0.017 ppb for I-CTX-1 equivalent toxicity may be considered. CFP is known in tropical regions for centuries, and it is an increasing risk of food poisoning worldwide; it occurs now in non-endemic areas due to international trade of fish and fish products and the expansion of the geographic ranges of dinoflagellates as a likely result of global warming [12,16,17]. Intoxication by CTXs may cause neurological, gastrointestinal, and cardiovascular symptoms depending on the amount and type of the toxin ingested [1,2,3,4,5,6,7,18], and occasionally in severe cases, CFP can be fatal [19,20]. P-CTX-1 possesses risk to human health at concentrations higher than 0.022–0.1 ng g^−1^ in fish flesh [8,21]. About 10,000 to 50,000 people suffer from the illness annually [1]; however, this is likely a substantial underestimate considering the incidence of non-reported cases from remote areas and non-diagnosis. Only 2–10% of CFP cases are estimated to be reported to health authorities [4]. Currently, there is no routine, rapid, reliable, and cost-effective point-of-care (POC) test that can detect ciguatoxins on-site or prior to consumption. Identification and quantification of CTXs is challenging even for laboratories due to the low CTX concentrations in fish flesh, the low recommended limit of 0.01 ng g^−1^, and the lack of reference materials and standards for all CTXs. The concentration of toxins in fish liver is about 10–50 times higher than in muscle tissue [22,23], thereby CFP becomes more problematic in communities consuming fish viscera. In extreme cases, such as for the liver of a large moray eel caught in Kiribati, toxicity can be as high as 539 ng g^‒1^, 50,000 times higher than the accepted safety level of 0.01 ng g^‒1^ [23]. The symptoms of CFP were first described by Captain James Cook and Don Antonio Parra in the 17th century during their exploration of the Pacific Ocean and Caribbean Sea, respectively [3,24]. CFP was finally linked to dinoflagellates in 1977 [25]. Several methods have been developed to test for CTX presence in fish, ranging from indigenous observations and animal mortality tests to modern analytical techniques. The present review aims to summarize such methods and identify future challenges in CFP testing. *Gambierdiscus* strains, from which *G. polynesiensis* in the Pacific Ocean and *G. excentricus* in the Atlantic Ocean represent the major threat to human health [26,27], are known to produce not only CTXs but other toxins, such as the water-soluble and structurally related maitotoxins [28,29] and gambierones [30,31]. However, the contributions of these latter toxins to CFP is insignificant compared to that of CTXs, due to their high water solubility and low oral potency [8,9]; therefore, these toxins are beyond the scope of this review.

## 2. Ciguatoxins

The metabolic modification of dinoflagellate toxins in fish produces a large number of structurally related CTX congeners. Multiple CTX congeners exist in fishes, and each may contribute to CFP. To date, 47 CTXs have been identified but less than half are structurally characterized due to the insufficient amounts of pure toxin available for analysis. Legrand et al. isolated 0.35 mg of pure P-CTX-1 from 125 kg of fish viscera, including 43 kg of liver, from 4150 kg of moray eels, *Gymnothorax javanicus* [32]. The specific chemical structures of major CTXs, however, were elucidated using NMR and mass spectrometry [33,34,35,36,37,38,39,40,41,42]. CTXs are composed of contiguous cyclic ether rings aligned in a ladder-like fashion, and the two termini of the rigid ladder are varied in congeners. Most of the CTX congeners possess a primary hydroxyl group that may allow selective derivatization. The toxicity of various CTX congeners are different. On the basis on their acute intraperitonial median lethal dose (LD_50_) in mice, EFSA has adopted the following toxicity equivalency factors (TEFs) for CTXs: P-CTX-1 = 1, P-CTX-2 = 0.3, P-CTX-3 = 0.3, P-CTX-3C = 0.2, 2,3-dihydroxy-P-CTX-3C = 0.1, 51-hydroxy-P-CTX-3C = 1, P-CTX-4A = 0.1, P-CTX-4B = 0.05, C-CTX-1 = 0.1 and C-CTX-2 = 0.3 [1].

**Figure 1:**
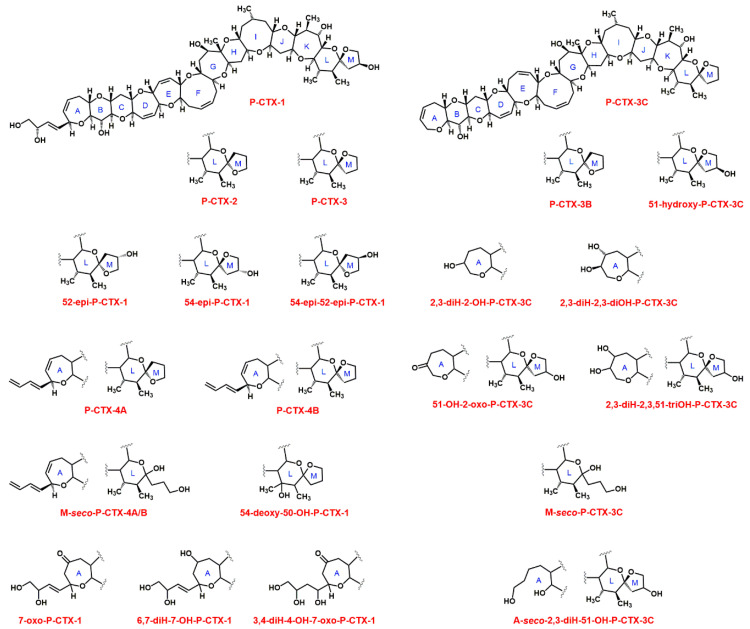
Structure of Pacific CTXs (alternative or old names are given in Table 1).

To date twenty-two CTXs have been identified from Pacific fish samples (Table 1). The skeletal structures of the 22 structurally characterized toxins can be separated into two groups, the P-CTX-1 (or CTX-1B) type and the P-CTX-3C type. P-CTX-1 (mass 1110.6 Da, C_60_H_86_O_19_) exhibits the highest toxicity against mice [33]. Molecular masses of P-CTXs are summarized in Table 1, and structures of CTXs are shown in Figure 1.

**Figure 2:**
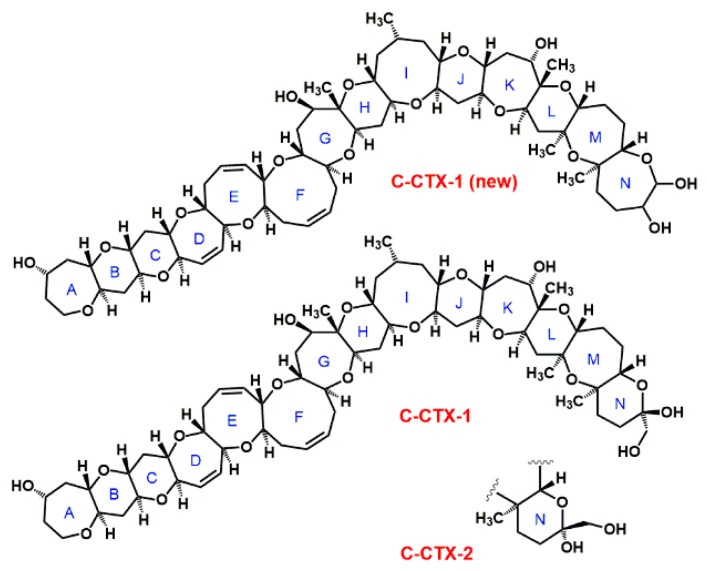
Structure of C-CTX-1 and C-CTX-2, according to Lewis et al. [35], and the suggested new structure, C-CTX-1(new) [36].

Twelve Caribbean CTXs have been identified thus far [34], and the structures of the two major toxins (epimers C-CTX-1 and C-CTX-2, mass 1040.6 Da, C_62_H_92_O_19_, see Figure 2) have been determined [35]. The molecular structure of C-CTX-1 has been recently revised [36] (Figure 2). Based on molecular fragmentation in a mass spectrometer at high collision energies, the N-ring of C-CTX-1 is more likely to be a seven-membered ring [36] than a six-membered [35]. Structure and toxicity of the other 10 CTXs have not been established yet. C-CTX-1 is considered to be 10-times less toxic than P-CTX-1 [8].

Six CTXs have been identified to date from fishes and sharks of the Indian Ocean [15,19,43], two isomer pairs with masses of 1140.6 Da (I-CTX-1 and -2, C_62_H_92_O_19_) and 1156.6 Da (I-CTX-3 and -4, C_62_H_92_O_20_) [15], as well as two congeners with only 2H less, 1138.6 Da (I-CTX-5, C_62_H_90_O_19_) and 1154.6 Da (I-CTX-6, C_62_H_90_O_20_), which corresponds to the formation of a double bond [19]. The exact structure of these toxins has not been determined as yet. Various experiments have indicated that the toxicity of both I-CTX-1 and -2 is 60% and both I-CTX-3 and -4 is 20% of the P-CTX-1 potency [15].

## 3. Extraction of Ciguatoxins from Fish Tissue

**Figure 3:**
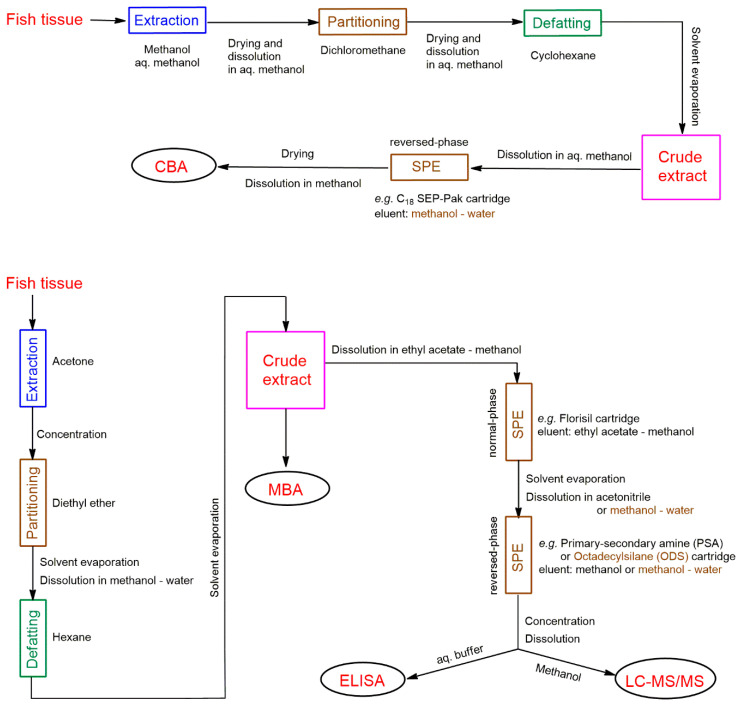
Examples of extraction of CTXs from fish tissue for various CTX tests [41,45,50,51].

Extracting CTXs from fish tissue is a critical step in CTX quantification and strongly influences analyte recovery and thus analytical reliability. This purification step is also important to efficiently remove matrix-derived interfering compounds that negatively affect sample analysis, such as lipids. Extraction methods involve multiple steps, and are time consuming; they include, in general, the following major steps: (1) extraction of raw, freeze-dried or cooked muscle tissue with a polar organic solvent (typically acetone or methanol); (2) purification of the extract by liquid-liquid partitioning (using diethyl ether, chloroform, or dichloromethane); (3) defeating the extract by liquid-liquid partitioning using hexane or cyclohexane; and (4) purification of the crude extract by solid-phase extraction (SPE), in one step using normal-phase or reverse-phase SPE, or more typically in two steps using consecutive orthogonal SPE phases. Several modified versions of the original extraction method of Lewis et al. [44] have been published during the last three decades, e.g., [12,43,45,46,47,48,49]; two currently used methods are summarized in Figure 3 as examples [41,45,50,51]. Varying the solvent used for extraction, the sample-to-solvent ratio, the number of extraction cycles used to extract fish tissue, and the number of SPE steps can influence the extraction efficiency of CTXs and extract purity, and determine time for extract preparation. A further complicating factor is that extraction efficiency depends also on the CTX analogue; for example, methanol is a good extraction solvent for various CTXs, but produces high levels of co-extractives. Using more polar solvent such as aqueous methanol limits the amount of co-extractives in the extract, is effective for the more polar CTX analogues, but much less effective for less polar CTX analogues [49]. Application of consecutive purification steps varies considerably among sample preparation protocols that exist in the literature. Most of the existing protocols are reviewed by Harwood et al. in 2017 [49]. Selection of the extraction protocol is not unambiguous, and unsurprisingly no validated extraction method exists to date. Current extraction protocols are complicated and not efficient enough.

The fish tissue extraction process due to several purification steps is slow compared to the time frame of modern analytical techniques (e.g., liquid chromatography-tandem mass spectrometry, LC-MS/MS, see below), therefore it is the rate-determining step for testing a fish sample. To decrease extraction time, a ciguatoxin rapid-extraction method (CREM), which uses only 2 g of fish tissue and combines the first three extraction steps mentioned above by applying a methanol-hexane mixture, has also been developed by Lewis et al. [52]. The method was updated and modified later by Stewart et al. [53] and Meyer at al. [54]. The rapid extraction method was estimated to be two-to-three times faster than the standard method for extraction and clean-up of CTXs [53]. It is, however, less effective than ‘normal’ protocols in terms of efficiency and toxin yield [54], and therefore not widely used by all specialized laboratories. Although, in principle, higher extract purity is better for all CTX detection techniques, it is worth considering minimum extract purity requirements for various analytical techniques, regarding time and cost. The crude extract is sufficient for mouse bioassay (MBA), the SPE provides sufficiently pure extract for Cell-based Assay (CBA), Receptor-binding Assays (RBA), Enzyme-Linked ImmunoSorbent Assay (ELISA) and LC-MS/MS. Further high-performance liquid chromatography (HPLC) separation produces purer fractions which can be useful for specific investigations, such as for example the determination of toxicity profile using CBA-N2a (see Section 4.4 below).

## 4. Detection and Quantification of Ciguatoxins

**Figure 4:**
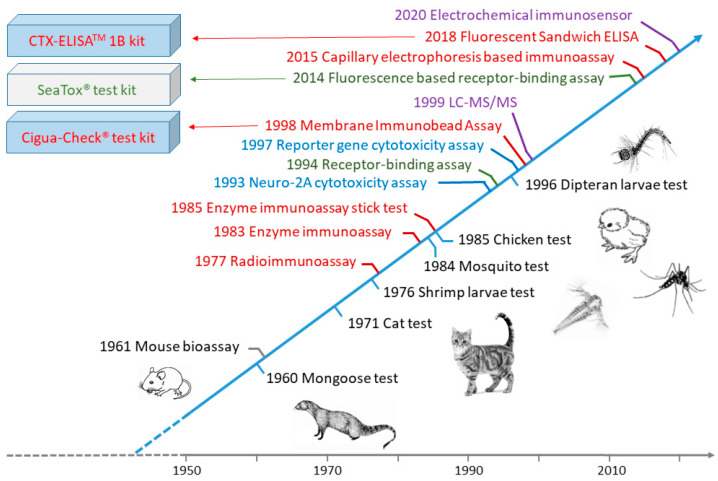
Timelines of CTX detection methods.

Due to the serious threat to human health caused by CFP, to date a wide variety of methods have been developed to detect CTXs in fish, including native tests, animal mortality tests, biological methods (cytotoxicity assays, receptor-binding assays and immunoassays), and chemical methods (HPLC with fluorescence detection, LC-MS/MS) (Figure 4). Many of these methods are not specific to CTXs, inadequate for quantification, or allow quantification of CTXs only with results expressed in “equivalent of a CTX standard”. Currently, the most advanced methods for monitoring CTXs are based on combination of biological and chemical methods into two steps by screening fish extract toxicity with sensitive functional assays first, followed by confirmation of the presence of CTXs via LC-MS/MS.

### 4.1. Indigenous Tests

Island communities that are strongly dependent on fish for food resources have developed various means over centuries to decrease the risk of CFP [10,11,55,56]; these include rubbing a small piece of liver on the mouth or skin and then testing for itchiness, cooking fish with a silver coin or copper wire and assessing discoloration, observing the color of fish gallbladder, examining food avoidance by ants and flies, feeding dogs, cats or pigs with suspected fish and observing sickness or fatality of animals, and bleeding and *rigor mortis* tests [10,11,55,56]. A fish is considered to be toxic in the bleeding test if haemorrhagic signs are visible at an incision on the tail of the dead fish. In the *rigor mortis* test, a fish is considered to be toxic if its flesh is flaccid an hour after death. All of these native and traditional test methods are now discredited due to their lack of specificity and the regular occurrence of both false negative and positive results. Darius et al. investigated the accuracy of the bleeding and *rigor mortis* tests by comparing test results with laboratory toxicity data obtained via the RBA and CBA on neuroblastoma cells [56], and concluded that intoxication in communities where CFP is highly prevalent may be reduced on the basis of traditional knowledge and a good understanding of high-risk versus relatively safe fishing areas.

### 4.2. Animal-Feeding Bioassay Tests

CTXs are toxic to a wide range of animal species [57]. Animal bioassay tests were developed during the 20th century. These tests were based on feeding cats [58], mongooses [59], chickens [60], or Dipteran larvae [61] with the flesh or viscera of suspected fish, or treating mosquitoes [62] or brine shrimp larvae [63] with fish extracts, and observing signs of intoxication and death of animals over time. Symptoms of cats and mongooses after being fed with toxic fish have been found to be similar in some respects to those of humans [59]. These reactions after a single test feeding, within 48 h, have been classified in five stages based on the maximal response of the test animal and numbered as: 0 = no symptoms; 1 = slight weakness and flexion of the forelimbs; 2 = slight motor ataxia, more pronounced flexion of the forelimbs, and weakness of the hind limbs; 3 = moderate motor ataxia with weakness and partial paralysis of limbs and body musculature; 4 = acute motor ataxia and extreme weakness or coma; and 5 = death. Stages 3–5 are indicative of high toxicity, 1–2 moderate toxicity, and 0 non-toxicity in fish. However, neither cats nor mongooses are satisfactory test animals to establish an LD_50_ because cats often regurgitate the test meal and mongooses commonly consume too much fish to permit the necessary replicate testing [59]. The chicken-feeding test is based on force feeding 8–10 days old chicks with cooked fish tissue (10% of body weight) and assessing the change in body weight of the study animals over a 48-h period [60]. The response of chickens to being fed contaminated fish liver has been found to be roughly quantitative. The mosquito bioassay test, where mosquitoes (*Aedes aegypti*) are intrathoracically injected, requires much smaller amounts of fish samples (8 g) than the tests discussed above, is much cheaper due to the low cost of mosquitoes, and is able to provide a LD_50_ value in 2 h [62]. This test requires, however, a fish extract, therefore use of laboratory, as it is not practical to conduct the extraction procedure under field conditions. Based on the mosquito bioassay and human symptomatology, the minimum lethal dose of P-CTX-1 in humans has been estimated to be 0.02 ng g^−1^ [64]. Although brine shrimp are seemingly unaffected by consuming finely ground ciguateric fish, their larvae have been found to be sensitive to fish extract. The brine shrimp larvae bioassay was based on treating approximately 100 freshly hatched larvae in artificial sea water with fish extract and observing the proportion of larvae that died over a 24-h period [63]. The Dipteran larvae-feeding test is extremely simple, does not require cooking or any pretreatment of fish samples, and can be evaluated visually; therefore, this test has been suggested to be appropriate for use in communities inhabiting remote islands that have no laboratory facilities [61]. The larvae, however, are sensitive to other toxic substances. In the Dipteran larvae test, ten larvae are placed on ca. 5 g of fish sample and the inhibition of larval growth is followed for 3−24 h. Fish samples containing more than 1 ng CTXs in 1 g of flesh kill the larvae in about 3 h. By weighing the larvae and comparing them to healthy reference samples, a limit of detection (LOD) of 0.15 ng g^−1^ can be achieved [61]. Although animal feeding tests, in general, are simple, easy to implement and do not require complex analytical equipment, such tests are insufficiently sensitive, incapable of providing specific information on individual toxins, time consuming, cannot be automated, and are expensive due to the required animal facilities and expertise. Further, there are serious ethical concerns about the application of these tests. Unsurprisingly, none of these tests currently are used in modern laboratories.

### 4.3. Mouse Bioassay (MBA)

**Figure 5:**
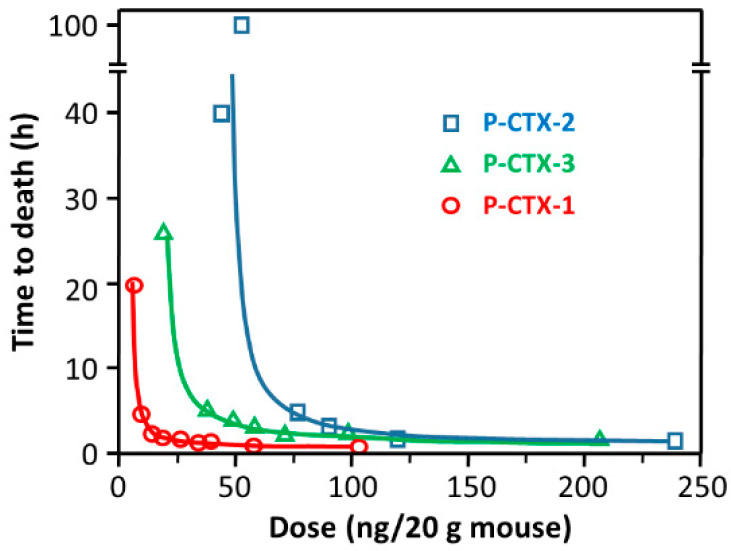
Relationship between intraperitoneal dose in mice and time to death for Pacific CTXs, reproduced with permission from [33]. Copyright Elsevier Ltd., 1991. The equation of the fitted curves is shown in the main text.

The mouse bioassay [65,66] is the only animal test today that remains in use, e.g., [67], despite its disadvantages and ethical concerns. The MBA is simple and does not require complex analytical equipment, but it is expensive due to the need for animal facilities, is time consuming, and cannot be automated. The MBA provides information only about the total toxicity of a sample, therefore lacks specificity, and CFP caused by CTXs may be overestimated. The limit of quantitation (LOQ) of MBA is approximately 0.56 ng g^−1^ for P-CTX-1 [1], therefore the bioassay is insufficiently sensitive to cover the suggested tolerance limit of 0.01 ng g^−1^ in fish. CTXs are highly potent toxins in mice by either the intraperitoneal (i.p.) or oral route [33]. Injecting fish extract intraperitoneally into mice is the generally applied method in MBA [1,3]. Raw extracts are usually suspended in 1% Tween 60, 0.9% saline solution prior to injection. The test is administered either by establishing dose/survival-time relationships or by observing mice for motor ataxia or other bodily dysfunction for 24 h after injecting serial dilutions of CTX extracts. The end point in the assay is the death of the test animal. The dose-vs-time-to-death relationships for CTXs (Figure 5) are found to be as follows: log(dose) = c log(1 + *t*^−1^), where dose is in mouse units (MU), time (*t*) to death is in hours, and constants c is 3.3, 2.4, 3.9, and 2.3 for pure P-CTX-1, pure P-CTX-2, pure P-CTX-3, and partially purified P-CTX, respectively. One MU is defined as the i.p. LD_50_ dose for a 20 g mouse, equal to 5.0, 46 and 18 ng P-CTX-1, PCTX-2, and P-CTX-3, respectively [33].

### 4.4. Cell-Based Assay (Cytotoxicity Assay)

Cell-based assays are dependent on the toxic activity of fish extracts on cultured cells and reflect the combined potency of related toxins in the mixture. The overall toxicological effects of CTXs are caused by the action of CTXs on neuronal potassium and voltage-gated sodium channels [2]. CTXs bind quasi-irreversibly to voltage-sensitive sodium channels, enhancing sodium influx into cells, causing them to open at the normal cell-resting membrane potential, thereby impeding normal function. In addition, CTXs also inhibit neuronal potassium channels, which is likely to act in concert with effects on voltage-gated sodium channels to increase neuronal excitability [2,3]. Evaluation of cell viability forms the basis of CTX determination in CBAs [1].

**Figure 6:**
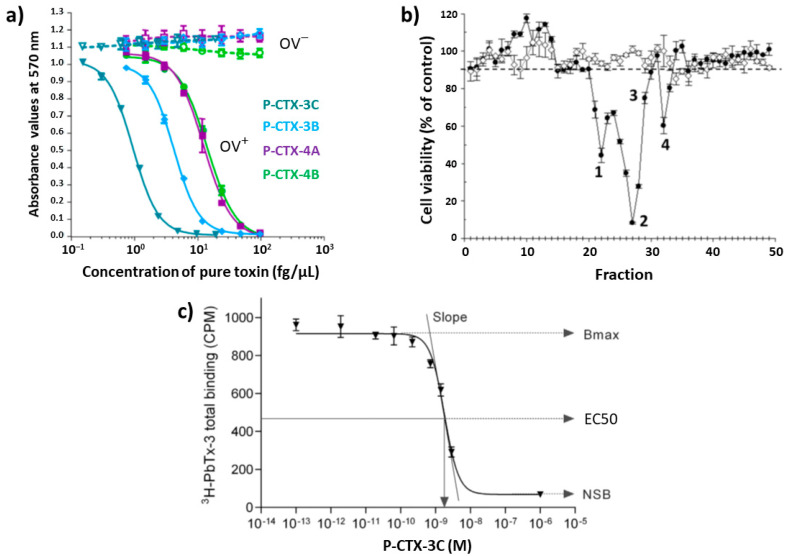
(**a**) Dose-response curve for N2a cells under OV^+^ (cells treated with ouabain and veratridine mixture) conditions and under OV^−^ (untreated cells without ouabain and veratridine added) conditions, exposed to increasing concentrations of four P-CTX standards, reproduced with permission from ref. [45]. Copyright MDPI AG, 2018; (**b**) CBA-N2a cytotoxicity profile of an HPLC fractionated sample, reproduced with permission from ref. [51]. Copyright MDPI AG, 2019; (**c**) Typical sigmoidal dose response curve of R-RBA. Bmax is the maximum binding of the bound radioligand (in counts per minute, CPM) in the absence of competing CTXs. Non-specific binding (NSB) represents the minimum total radioligand binding in the presence of saturating concentrations of CTXs, the EC50 is the effective concentration at 50%, reproduced with permission from [80]. Copyright Elsevier Ltd., 2018.

Several cell and tissue-based assays were developed previously, namely the guinea pig ileum [68], guinea pig atrium [69,70], isolated frog nerve fiber [71], crayfish nerve cord [72], and blood cell hemolytic [73] tests. These tests, however, are outperformed by the mouse neuroblastoma cell assay (CBA-N2a) and thus are no longer employed. The CBA-N2a is widely used today, and its development by Manger et al. [74] is one of the most important milestones of replacing MBA in modern laboratories. The assay is based on the colorimetric detection of metabolically active N2a cells treated with CTX extract in the presence of ouabain/veratridine [74,75,76]; it expresses negatively the concentration of various voltage-gated sodium channel toxins and assesses cell death [3]. CTXs have no cytotoxic effect on N2a cells, therefore their detection requires addition of veratridine (a sodium-channel-activator that have a different binding site than CTXs) and ouabain (a sodium/potassium ion ATPase inhibitor). The combined effect of CTXs together with ouabain and veratridine causes an elevation of intracellular sodium ions to toxic levels in cells and a resultant decrease in cell viability that can be measured as a function of CTX concentration. Toxins are detected as a dose-dependent loss of cell viability, based on an end-point determination of mitochondrial dehydrogenase activity, due to the synergistic effect of ouabain/veratridine-induced cytotoxicity by CTXs (Figure 6a). Color development is based on the ability of active cells to reduce 3-[4,5-dimethylthiazol-2-yl]-2,5-diphenyltetrazolium (MTT) to a blue-colored formazan product. The advantage of this MTT-based bioassay is that it is more sensitive for CTXs (at ng/g fish level) than the mouse bioassay, and suits to automation due to color reading [1]. Results obtained from CBA-N2a bioassays of fish extracts have correlated well with those obtained from MBA [1,74]. Although CBA-N2a have been widely used in the last three decades and the protocol has undergone numerous changes [77], a consensus assay protocol is still lacking. To this end, research to standardize this CBA [77,78] and to avoid matrix effects [79] also have been conducted. Viallon et al. revisited recently the CBA-N2a assay by investigating six key parameters, namely cell seeding densities, cell layer viability after 26 h growth, MTT incubation time, veratridine and ouabain treatment, and solvent and matrix effects [77]. A step-by-step protocol was defined by identifying five viability controls for the validation of CBA-N2a results, therefore, the improved method is an important step towards implementation of a reference detection test.

An advantage of the CBA-N2a assay is that necessary materials and reagents, as well as basic laboratory equipment are commercially available. A major disadvantage of CBA-N2a, however, is that it is time consuming, including, in general, 24 h incubation of neuro-2a cells, 24 h exposure of the neuro-2a cells to fish extracts, and a 4–6 h cell viability assessment. Achieved LOD and LOQ using this assay depends also on assay protocol. Nonetheless, CBA-N2a is able to provide LOD below the clinically relevant toxin levels in fish tissue. Selected examples for CBA application are summarized in Table 2 (more data are provided in the cited articles).

Fairey et al. modified the cell-based directed cytotoxicity assay and developed a reporter-gene modification by using CBA-N2a clones expressing c-*fos*–Luciferase; the assay was thereby utilizing a luciferase-catalyzed light generation as an endpoint and a microplate luminometer for quantification [81,82]. c-*fos* is an immediate response gene and a sensitive biomarker to localize the effects of toxins. This assay, however, is not commonly used possibly due to the problematic interpretation of the bell-shaped dose–response curve and the cost of fluorescent dye.

N2a cell lines are widely and routinely used in cell-based assays. However, other cell lines have also been tested for potential application in CBA. Zimmermann et al. [83] and Lewis et al. [84] developed fluorescent CTX assays using the human neuroblastoma cell line SH-SY5Y, expressing a range of voltage-gated sodium channel subtypes. SH-SY5Y cells were loaded with Calcium-4 No-Wash dye absorbed into the cells’ cytoplasm. Cells were incubated for 5 min with CTXs prior to addition of veratridine. Fluorescence responses to CTXs, arising due to calcium ion influx into cells, were recorded using a Fluorescent Imaging Plate Reader. The performance of the SH-SY5Y assay was comparable to a N2a-based cytotoxicity assay [84]. The assay, however, is currently not widely used because it requires specialized equipment, the fluorescent dyes are expensive, and a small carryover of maitotoxins into the CTX fraction during purification steps could potentially obscure CTX responses due to saturation of fluorescence by maitotoxin-induced increase of intracellular calcium ions [3].

**Table 2:** Examples for the application of cytotoxicity and receptor-binding assays ^1^.

Assay ^1^	Fish Species (Family)	Equiv. ^2^	Mass ^3^ (g)	Conc. ^2^ (ng g^−1^)	LOQ ^4,5^ (ng g^−1^)	Ref.
CBA-N2a	*Carcharhinus leucas* (Carcharhinidae)	P-CTX-1	10 ^6^	92.78	0.13	[19]
CBA-N2a	*Lutjanus sp.* (Lutjanidae)	P-CTX-1	5	0.4708	0.032	[21]
CBA-N2a	*Gymnothorax spp.* (Muraenidae)	P-CTX-1	20 ^7^	539	0.0016	[23]
CBA-N2a	*Seriola fasciata* (Carangidae)	C-CTX-1	15	1.4	n.a.	[51]
CBA-N2a	*Chlorurus microrhinos* (Parrotfish)	P-CTX-3C	10	6.66	0.064	[77]
CBA-N2a	*Epinephelus merra* (Serranidae)	P-CTX-3C	10	3.31	0.064	[77]
CBA-N2a	*Seriola fasciata* (Carangidae)	P-CTX-1	10	6.231	0.0096	[78]
CBA-N2a	*Balistes vetula* (Balistidae)	C-CTX-1	n.a.	0–0.11	0.006	[85]
CBA-N2a	*Sphyraena barracuda* (Sphyraenidae)	C-CTX-1	10 ^7^	2.1	0.039	[86]
CBA-N2a	*Balistapus undulatus* (Balistidae)	P-CTX-1	5	4.64	0.00195	[87]
CBA-N2a	*Epinephelus multinotatus* (Serranidae)	P-CTX-1	5	6.49	0.00195	[87]
CBA-N2a	*Sphyraena barracuda* (Sphyraenidae)	C-CTX-1	n.a.	0.099	0.001	[88]
R-RBA	*Scarus altipinnis* (Scaridae)	P-CTX-3C	5	0.36–4.52	0.155	[89]
R-RBA	*Kyphosus cinerascens* (Kyphosidae)	P-CTX-3C	5	0.46–4.25	0.155	[89]
R-RBA	*Plectropomus leopardus* (Serranidae)	P-CTX-3C	5	0.36–3.29	0.155	[89]
R-RBA	*Liza vaigiensis* (Mugilidae)	P-CTX-3C	5	16.23	0.155	[89]
F-RBA	*Pterois volitans* (Scorpaenidae)	C-CTX-1	5	0.1–0.2	0.23	[90]

^1^ CBA-N2a = mouse neuroblastoma cell assay; RBA = receptor binding assay (R-radioligand-based, F-fluorescence-based); n.a. = not available; ^2^ Composite toxicity (ng C-CTX-1 (TEF = 0.1), P-CTX-3C (TEF = 0.2) or P-CTX-1 toxin equivalent/g fish tissue); ^3^ Mass of fish tissue used for the analysis; ^4^ LOQ for CBA-N2a: (EC_50_ of P-CTX-3C/maximum concentration of dry extract)×(dry extract weight/fresh weight of flesh tissue extracted); ^5^ LOQ determination for RBA: LOQ = IC_50_ of P-CTX-3C/quantity maximum of a sample that does not cause matrix interferences; ^6^ Shark stomach sample; ^7^ Liver.

CTXs accumulate in fish tissue due to their lipophilic character, however, they also circulate in blood for some time. Bottein Dechraoui et al. were able to detect CTX in blood of mice after 12 h post-exposure of sublethal dose of Caribbean ciguatoxic extract (0.59 ng g^−1^ C-CTX-1 equivalents), and pointed out that neuroblastoma assay (LOD 0.006 ng ml^−1^ C-CTX-1) is suitable to monitor CTX in blood at sublethal doses in mice and argued that CTX monitoring in blood could be a useful procedure for fish screening [91]. Blood is a much simpler matrix than fish tissue, and CTX recovery from fresh blood is close to 100% due to lack of matrix effects [92]. Taking blood samples is a non-lethal sampling method for detection of CTXs in wild fish. O’Toole et al. studied the toxin level in tissue, blood and liver of the great barracuda (*Sphyraena barracuda*) [88] and observed a correlation between blood and liver toxin concentrations.

Although cytotoxicity assays are simple and provide an alternative to the MBA, they are currently not time- and cost-effective for mass screening, reflect only the combined potency of all-sodium-channel blocking toxins in an extract, and thus fail to provide any information about the toxin profile. Considering this latter point, however, limited information can be obtained by fractionating an extract using HPLC and then testing the toxicity of these fractions. Estevez et al. fractionated an amberjack sample, and CBA-N2a toxicity profile indicated the presence of at least four toxins (Figure 6b), which was confirmed by LC-MS/MS [51].

### 4.5. Receptor-Binding Assays (RBA)

CTXs compete with brevetoxin for the same neurotoxin receptor sites at sodium channels, having at least 20–50-times higher affinity [81], therefore CTXs are competitive inhibitors of brevetoxin binding [7,33,93,94]. Measuring the inhibition of radioactively labeled [3H]-brevetoxin-3 binding to rat brain membrane can be used to detect CTXs in fish extracts using either test tube [89,95] or microplate format [96]. Radioligand RBA (R-RBA) is based on competitive binding between CTXs and a radioactively labeled brevetoxin for a given number of available receptor sites in a membrane preparation. When the concentrations of the radioactively labeled brevetoxin and the receptor are kept constant and the concentration of CTXs increases, the binding of the radioactively labeled brevetoxin to the receptor sites is proportionally reduced. A competition dose-response curve can be constructed by measuring the concentration of the radioligand-receptor complex across a range of concentrations of CTX standard (Figure 6c), and the amount of CTXs in an unknown sample can be quantified [80]. RBA and CBA-N2a are more specific to CTXs than the MBA due to receptor binding, but RBA and CBA-N2a, like the MBA, do not provide any information on the toxin profile. The sensitivity of RBA is highly dependent on the receptor source. Bottein Dechraoui et al. compared the performance of the R-RBA and the CBA-N2a cytotoxicity assay, and the RBA was found to be 12-fold less sensitive than CBA-N2a for Caribbean ciguatoxin analysis [86]. It was also noted that R-RBA provided systematically higher estimates of CTX concentration than CBA-N2a [86,97]. Although R-RBA have been widely used, screening methods have not been standardized. Díaz-Asencio et al. recently published a methodological development and also determined criteria for quality control [80]. The developed microplate format was able to detect 0.75 ng g^−1^ P-CTX-3C equivalent in fish tissue in only 3 h (process for full plate, not considering up to 2 d extraction time for 10 samples). An analyst could run an estimated 32 samples per day (with up to eight samples per plate run in triplicate at two dilutions) [80].

Large-scale field screening using R-RBA was performed by Darius et al. while evaluating toxic versus safe areas at two sites in French Polynesia, using the R-RBA test-tube format and rat brain synaptosomes as receptors [89]. Results indicated significant disagreement with the knowledge of local people regarding toxicity in several cases and findings were congruent only for fish species with high risk of CTX accumulation, indicating the need of close monitoring to prevent epidemiological outbreaks of CFP (see examples in Table 2, and more data in the original reference).

A disadvantage in the original radioligand format, and a constraint to application, is that R-RBA requires application of radioactive [3H]-brevetoxin compounds. To overcome this problem, McCall et al. recently developed a fluorescence-based RBA method (F-RBA) [98], which assesses competitive binding between CTXs and fluorescently labeled brevetoxin-2. Several fluorescent compounds, including BODIPY, 6-TAMRA, Texas Red, Alexa Fluor 350, Fluorescein, Coumarin, and Dansyl hydrazine, were conjugated to polyether brevetoxin-2, from which the BODIPY-brevetoxin-2 exhibited the best performance in terms of lowest nonspecific binding. The constructed F-RBA was faster (with an assay and analysis time of less than 3 h versus overnight), less expensive and safer than R-RBA, and generated binding constants comparable to the radioligand assay. As a continuation of this work, Hardison et al. developed a F-RBA using fluorescently labeled BODIPY^®^-brevetoxin-2 [97]. The method was relatively fast and took approximately 2 h to perform and exhibited a LOD and LOQ of 0.075 and 0.1 ng g^−1^ P-CTX-3C equivalent, respectively. Based on this principle, a commercial test kit for detecting CTXs was developed by SeaTox Research Inc. (Wilmington, NC, USA; https://www.seatoxresearch.com/testing-kits/) and currently distributed by MARBIONC^®^ Development Group LLC (Wilmington, NC, USA; http://www.marbionc.org/gallery/detail.aspx?id=2274946). The SeaTox kit can be used as a screening or quantitative tool, and CTX analyses can be completed in less than two hours after fish tissue extraction.

### 4.6. Immunoassays

Immunoassays are dependent on the application of a high-affinity antibody that is selective and specific for CTXs, therefore, CTX detection and quantification with immunoassays are based on the structure of CTXs, not on their toxicity. In principle, a 100%-specific antibody could capture only one target, therefore detection of all toxins and toxin profile determination of a mixture of different toxins would require application of the same number of specific antibodies than the number of targets. If the antibody is less specific and selective only for a common structural motive of target molecules, it can capture several targets, but cross-reactivity with non-target compounds having the same structural element cannot be avoided. Production of antibodies is one of the key issues and constraints of immunoassays. In all cases, a label is attached to the antibody to detect target-antibody interaction; this label can be a radioisotope, enzyme, or fluorescent probe. Immunoassay methods, in general, are fast and easy to use, the sole exception being the radioimmunoassay (RIA). Unless several antibodies are used, they do not provide information on the toxin profile. Selecting antibodies only against the major toxin might also be problematic; P-CTX-1 in carrier fish, for example, represents in many cases greater than 90% of the toxins, but C-CTX-1 in fish represents only about 50% of total toxins [8]. Another problem that may rise with immunoassays is the possible cross-reaction of low-potency CTXs with antibodies, which increases the possibility of obtaining false-positive results. Antibodies developed for Pacific CTXs may not be suitable for testing Caribic CTXs, and *vice versa*.

**Figure 7:**
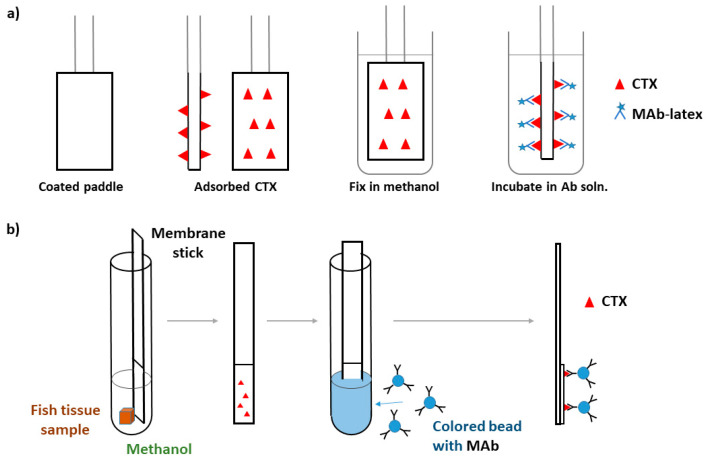
(**a**) Working principle of the simplified solid-phase colored latex immunobead assay (SPIA) test, adapted from ref. [107]; (**b**) Working principle of Membrane Immunobead Assay (MIA) test, adapted from [112].

The first immunoassay test, RIA, was developed by Hokama et al. in 1977 to assess fish tissues directly [99]. Labeled CTX antibodies were prepared by: (1) coupling purified CTX, isolated from toxic eel tissues, to human serum albumin; (2) injecting the CTX-human serum albumin conjugate into sheep for generating the anti-CTX-human serum albumin; (3) bleeding animals and collecting serum after 8 weeks; and (4) iodinating sheep anti-CTX-human serum albumin with ^125^I. This method was successfully used in the following years to test and reject toxic fish on the Hawaiian market [100], but it was time-consuming, expensive, and required special radioisotopic facilities, therefore it was impractical for routine screening of fish samples. The same sheep anti-CTX serum synthesized for RIA was used by Hokama et al. to develop an enzyme-immunoassay (EIA) by coupling horseradish peroxidase (HRP) to sheep anti-CTX-human serum albumin instead of ^125^I [101]. 4-chloro-l-naphthol was used as substrate for the enzymatic reaction with spectrophotometric reading. EIA exhibited similar sensitivity and specificity to that of the RIA, yet, it was easier to run and economically feasible for screening fishes. However, this test remained labor intensive, and cross-reactions occurred with other polyether compounds. As a further development, Hokama constructed the first POC test utilizing the same sheep-anti-CTX antiserum [102,103]. The simplified enzyme immunoassay stick test (S-EIA) was rapid and did not require any instrumentation. The reagents for S-EIA were similar to those of EIA, and the salient feature of S-EIA was use of a coating (Liquid Paper, Liquid Paper Corp., Rockville, MD, USA) applied to a bamboo stick to adsorb the lipid CTX and its related polyether toxins onto the stick. Fish samples were poked with the stick and after fixation, the stick was immersed consecutively into sheep-anti-CTX-HRP solution and substrate solution. The substrate color change was read after 10 min, which provided a fast estimation of toxin content as being negative, borderline, or positive. All of these methods – RIA, EIA, and S-EIA—used fish flesh directly without extraction, but the antibody was not sufficiently selective, as the polyclonal sheep-anti-CTX detected not only CTXs but also structurally-related polyether toxins, including okadaic acid and brevetoxin [104,105]. This attribute resulted in false-positive tests [8,10]. Lack of sufficient sensitivity for S-EIA was also noted [106]. Due to these drawbacks, Hokama et al. prepared and used monoclonal antibodies, MAb, to CTXs in immunoassays [105]. Monoclonal antibodies are more selective to CTXs than are polyclonal variants, having some cross-reactivity with okadaic acid (16%), but little or none with polyethers (e.g., ionomycin). Using MAbs, Y. Hokama developed a simplified solid-phase colored latex immunobead assay (SPIA) for the field detection of CTXs and related polyethers (Figure 7) [107,108]. MAb-CTX was labeled with colored latex. In the simplified procedure, a paddle end of the stick coated with correction fluid (organic base solvent) is inserted into an incision in the fish so that the fish tissue is touched, then removed; the paddle end of the stick is then dried, fixed with methanol, and immersed into MAb-CTX-latex suspension. Visually detectable coloration of the stick is considered to be a positive test result. Hawaii Chemtect International (Pasadena, CA, USA) purchased the patents covering the S-EIA and SPIA tests and commercialized a rapid solid-phase immunobead assay (Ciguatect™) for the detection of ciguatera toxins [109]. The Ciguatect™ test kit could determine the ciguatera potential directly in the fish flesh or after toxin extraction. Toxin extraction increased the sensitivity and decreased the limit of detection [109]. The Ciguatect™ test was more sensitive than S-EIA and better suited to field applications [110]. (Note that the test is no longer used.) The S-EIA test required application of six assay sticks per fish [102,111].

Based on the same immunological principles as the SPIA, Hokama et al. developed a Membrane Immunobead Assay (MIA) using a polyvinylidene fluoride hydrophobic membrane laminated onto a solid plastic support to collect CTXs and a MAb to purified moray eel (*Muraenidae*) ciguatoxin attached to colored polystyrene beads [112]. Application of the hydrophobic membrane was an advantage in reducing non-specific binding of the immunobeads. The test procedure involved placing about 5 mg of fish tissue sample, 0.5 mL methanol, and the membrane stick into a test tube for 20 min, then removing, drying, and immersing this into 0.5 mL immunobead suspension (Figure 7). The intensity of the color on the membrane related to the concentration of the CTX. MIA exhibited much higher specificity than RIA, S-EIA, and SPIA [112]. Oceanit Test Systems, Inc. (Honolulu, HI, USA) marketed a POC test kit, Cigua-Check^®^ test kit, what was based on MIA for P-CTX-1, and developed to test rice-grain-size amounts of fish flesh and to detect CTX higher than 0.05 ng g^−1^ flesh [8]. In principle, this method was able to detect ciguatoxin at concentrations that induce clinical symptoms in humans (above 0.08 ng P-CTX-1/g fish flesh [8]). Concerns were, however, raised against the method’s sensitivity and specificity, as well as the interpretation of the test-strip results [113], and possibly due to these and marketing issues, this method is currently not commercially available.

**Figure 8:**
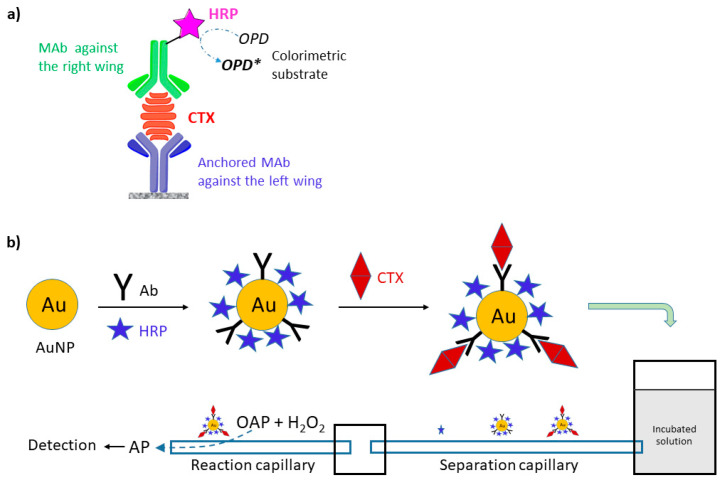
(**a**) Schematic of the sandwich ELISA detection of CTXs (*OPD* = *o*-phenylenediamine); (**b**) Illustration of the preparation of noncompetitive immunoassay with CE separation, adapted from [121].

Antibodies for antibody-based immunoassays are typically produced using scarce natural toxins. This limitation, in principle, could be overcome by replacing natural toxins with synthesized toxins, or with fragments of CTXs. The synthesis of fragments is more simple and cost-effective than that of the whole CTX molecule. These fragments, typically haptens, can be used to generate monoclonal antibodies against CTXs. Sandwich-type ELISA is very promising for increasing selectivity of assays by using two antibodies for the recognition of CTX, where one recognizes the left and the other the right wing of CTX. One of the MAb is conjugated with the enzyme label, typically HRP (Figure 8). To improve performance of immunoassays, Campora et al. constructed a sandwich-type ELISA, utilizing two antibodies, a chicken immunoglobulin Y specific to the ABCD domain of P-CTX-1 and a mouse monoclonal immunoglobulin G‒HRP conjugate label specific to the JKLM domain of P-CTX-1 produced by injecting chicken and mouse with synthesized CTX fragments [114]. Significant cross reactivity was not observed for brevetoxin-3, okadaic acid, or domoic acid. Good correlation was observed between this ELISA and CBA-N2a assays by screening three fish species commonly implicated in ciguatera fish poisoning in Hawaii [115]. Tsumuraya et al. developed sandwich-type ELISA detection protocols for the four principal congeners of Pacific ciguatoxins, P-CTX-1, P-CTX-3, P-CTX-3C, and 51-hydroxy-P-CTX-3C, using MAbs produced by immunizing mice with the corresponding left and right wing haptens [116,117,118,119]. P-CTX-1 could be detected specifically as low as 0.28 ng mL^−1^ without cross-reactivity with other related marine toxins; this concentration was still higher than the regulatory limit of 0.01 ppb [117]. As a further development, Tsumuraya et al. combined these MAbs into a single sandwich ELISA assay to detect any of these four CTX congeners [50,120]. Detection of the immunoreaction was changed to a fluorescent method using alkaline phosphatase(ALP)-linked MAb and a fluorescent substrate system, 2′-(2-benzothiazoyl)-6′-hyrodxybenzothiazole phosphate (BBTP). The fluorescent ELISA was highly sensitive, having a detection limit of less than 1 pg mL^−1^. P-CTX-1 spiked into fish at the recommended safety level of 0.01 ppb P-CTX-1 equivalent was reliably detected by this ELISA. The ELISA assay was shown to be very sensitive to CTXs, but required fish extract and laboratory conditions. Based on this ELISA, a fluorescent sandwich ELISA kit “CTX-ELISATM 1B” developed for detection of the P-CTX-1 series (P-CTX-1 and P-CTX-3) was commercialized and could be purchased from Fujifilm Wako Corporation (Osaka, Japan; cat. 382-14341) [120].

Zhang et al. developed a capillary electrophoresis (CE)-based immunoassay for detection of P-CTX-1, applying electrochemical detection and gold nanoparticles (AuNPs) as carriers of HRP and CTX antibodies (Ab-AuNP-HRP) as target-capture elements [121]. Crude fish extract was sufficient for detection, which involved mixing the extract with Ab-AuNP-HRP probe solution, dilution, incubation, and analysis by CE separation and electrochemical detection. The unbound HRP, Ab-AuNP-HRP probe and the formed CTX-Ab-AuNP-HRP immunocomplex were separated according to the velocity difference in the separation capillary, and catalyzed the o-aminophenol (OAP) and hydrogen peroxide reaction to 2-aminophenoxazine-3-one (AP) (Figure 8). This latter was electrochemically detected. Due to the high separation power of CE and the high target specificity of the immunoassay, a LOD of 0.045 ng mL^−1^ was achieved. As a further development, Zhang et al. fabricated an ultrasensitive immunoassay for P-CTX-3C detection exhibiting a LOD of 0.09 pg mL^−1^, based on CE separation and on-line sandwich immunoassay with rotating magnetic field [122]. The sandwich system utilized rabbit anti-P-CTX-3C-functionalized magnetite NP beads as immunosensing probes, and HRP and monoclonal sheep anti-P-CTX-3C-functionalized AuNPs as recognition elements. The rotating magnetic field enhanced the mixing efficiency and molecular binding rates, and the immunoreaction time of the assay was decreased to 15 min. The latter was much faster than normal ELISA.

The first electrochemical immunosensor for CTX detection was constructed by Leonardo et al. this year, 2020 [67]. Capture antibodies were prepared by immobilizing two different mouse MAbs on magnetic beads; these MAbs were able to bind to the left wing of P-CTX-1/P-CTX-3 and P-CTX-3C/51-hydroxy-P-CTX-3C, respectively. A mouse MAb, which was able to bind to the right wing of P-CTX-1, P-CTX-3, P-CTX-3C, or 51-hydroxy-P-CTX-3C, was biotinylated, linked to polyHRP-streptavidin signal reporter, and used as a detector antibody.

To develop the biosensor, the magnetic immunocomplexes were deposited on eight-electrode arrays, and the assay was performed by successively incubating the magnetic immunocomplexes with the CTX analyte and detector antibody. Although LOD and LOQ values of this CTX biosensor were higher than those of fluorescence ELISA, the electrochemical biosensor had an advantage of lower cost, the possibility to be integrated into compact analytical devices, and portability. The immunosensor was successfully applied to the analysis of fish samples and was able to detect P-CTX-1 at 0.01 ng g^−1^ toxin level, as well as exhibited a good correlation with CTX levels determined by the CBA-N2a.

### 4.7. HPLC, LC-MS/MS and LC-HRMS

CFP is a complex disease in which several different compounds contribute to the toxicity of a fish sample. Current CBA-N2a, F-RBA, and immunoassays are very sensitive, but do not provide information about the toxin profile. Unfortunately, CTXs do not have characteristic functional groups for spectroscopic detection, therefore separating and separately identifying toxins is the only viable route for toxin profiles, and HPLC is the key method for separating CTX congeners. Classical HPLC methods using UV detectors, however, are not sensitive enough to detect very small concentrations of CTXs at clinically relevant levels, and CTXs cannot be distinguished using UV detection due to the lack of useful UV chromophores [12,33,123,124]. At a terminus of the molecule, however, most of the CTX congeners have a primary hydroxyl group (see Figure 1 and Figure 2) that is available for fluorescence labelling. HPLC with fluorescence detection using 1-anthrylcarbocyanide and carbonyl azides or carbonyl nitriles of coumarin derivatives as labels was found to be more sensitive for CTX detection than HPLC-UV, however, still could not reach the recommended tolerance level of 0.01 ng g^−1^ for P-CTX-1 [12,125,126]. The main limitation of this technique was that it could not detect CTXs without a primary hydroxyl group (e.g., P-CTX-3C; see Figure 1), what is especially problematic for herbivores where CTXs without primary hydroxyl group are more abundant [127].

To utilize the separating power of HPLC, a sensitive detector is required. HPLC coupled with tandem mass spectrometry (HPLC-MS/MS) were applied for CTX detection the first time by Lewis and Jones in 1994 [128], and the first successful application of the combined HPLC-MS/MS technique for CTX detection at clinically relevant levels, with detection limits of at least 0.04 ppb for P-CTX-1 and 0.1 ppb for C-CTX-1, was reported by Lewis et al. in 1999 using gradient reversed-phase HPLC and an electrospray triple quadrupole mass spectrometer [129]. The method was significantly more sensitive than fluorometric HPLC. In the following decades LC-MS/MS became one of the leading and indispensable techniques of modern CFP laboratories due to the method’s ability to separate and identify toxins, and its uniqueness in providing toxin profiles. Both the identification and quantitation of CTXs, however, require reference toxin molecules. Although the molecular peak and fragmentation pattern in MS can provide valuable information for CTX identification, a diagnostic fragmentation pattern is not always achieved (water losses are often the most abundant fragment ions and depending on the MS instrument used, results may be different). LC-MS/MS must be used in combination to biological assay as a confirmation. In general, raw fish extract could be used for LC-MS/MS, but to decrease possible interference of lipids and fatty acids in fish tissue, the raw extract is usually purified using SPE in one or two steps (Figure 3). The presence of matrix co-extractives significantly interferes with ionization and causes severe signal suppression [49]. To reach very low detection limits, both HPLC and MS conditions must be optimized for sensitivity and selectivity, namely: LC conditions, ionization sources, ion choices and acquisition modes [41,46,130,131]; a comparison of analytical protocols to find the best conditions for sensitivity and/or selectivity for LC-MS/MS has been published recently [130].

**Figure 9:**
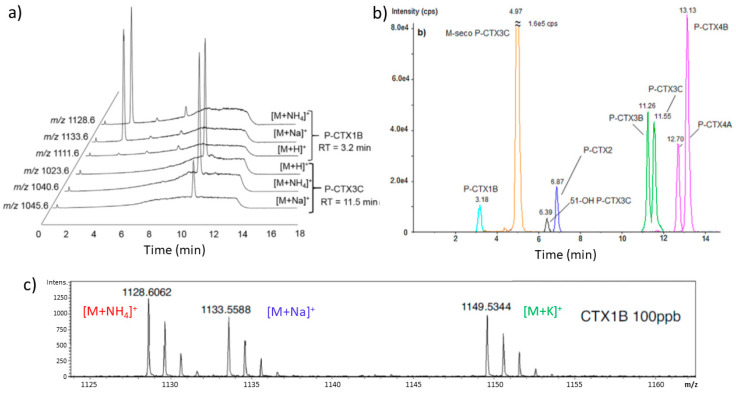
(**a**) LC-MS/MS chromatogram of standard CTX solutions showing that the [M + Na]^+^ and [M + NH_4_]^+^ ions were favored for P-CTX1B (P-CTX-1), while the [M + NH_4_]^+^ and [M+H]^+^ ions were dominant with similar intensities for P-CTX3C (P-CTX-3C) at identical conditions. A linear gradient of water-methanol solvent mixture, containing ammonium formate and formic acid, was used for chromatographic separation. SIM was performed in positive mode for ions at *m*/*z* [M + Na]^+^, [M + H]^+^ and [M+NH_4_]^+^. Reproduced with permission from ref. [130]; (**b**) LC-MS/MS chromatogram and retention time of P-CTXs standards using a Zorbax C18 column, water and methanol as eluents, and a linear gradient. The separation of various CTX congeners is shown. Reproduced with permission from [130]. Copyright Elsevier Ltd., 2018; (**c**) LC-HRMS high resolution mass spectra of CTX1B (P-CTX-1) obtained from CTX standard, showing adduct peaks and isotopic patterns, reproduced with permission from [132]. Copyright Elsevier Ltd., 2017.

LC separation is most frequently performed with acetonitrile-water or methanol-water linear gradients; the methanol-based mobile phases have been found to be more advantageous. Electrospray ionization and positive ion detection mode, using either Single Ion Monitoring (SIM), Multiple Reaction Monitoring (MRM) or Enhanced Product Ion (EPI) scanning, are typically used in MS. Applying MRM is advantageous for increasing specificity. The mobile phase composition, solvents and additives, and flow rate of the mobile phase affect ionization efficiency and ion abundance, therefore they have to be optimized. Monitoring ammonium or sodium adduct as the parent ion often exhibited higher signal-to-noise ratios than protonated parent ion; however, this depends not only on LC and MS conditions, but also on the analyzed CTX (Figure 9a). With formic acid, as a mobile phase additive, the sodium adduct was favored using either acetonitrile/water or methanol/water linear gradients in LC. The sodium adduct, however, has been found to be relatively stable, therefore to obtain MS/MS fragmentation its generation should be avoided by proper selection of eluent and additives, e.g., ammonium formate [130]. The ammoniated adduct ion, as a precursor ion, showed an advantage for selectivity through confirmatory transitions [130]. Figure 9b shows an example for the LC separation and detection of a mixture of CTX standards. The technique using triple quadrupole detectors (low resolution, LC-MS/MS) is significantly more sensitive than high-resolution MS coupled to HPLC (LC-HRMS, time-of-flight and orbitrap based spectrometers). An important advantage of HRMS, however, is that HRMS provides molecular formula and isotopic patterns of molecules (Figure 9c), therefore providing improved identification of CTX analogues [130,132]. LC-HRMS together with reference material may be the best choice for accurate identification of CTXs. The two techniques may be considered complementary, as LC-MS/MS is more adequate for quantitation and LC-HRMS performs better for identification of toxins. Selected examples for the application of LC-MS for CTX quantification in fish tissue, including fish species, toxin content and LOQ, are shown in Table 3 (more data are provided in the cited articles). Fish blood for determining the CTX content was studied recently by Mak et al. [133], and the matrix effect was found to be smaller than that of using fish muscle tissue. The main advantage of LC-MS is that it is very specific and adequately sensitive to detect CTXs at the clinically relevant toxin levels, and superior to any other techniques as the toxin confirmatory method.

## 5. Outlook and Conclusions

CFP is an old problem for communities in tropical regions that rely on seafood for survival, and CFP has now become a global issue due to the worldwide seafood trade, international travel, and ongoing expansion of the geographic ranges of fish contaminated with ciguatoxins. There is, therefore, high demand to test fish for CTXs prior to human consumption, both for mitigation of the health risks of CFP and for clinical identification of toxins in cases of intoxication. Currently, however, reliable biomarkers that can confirm exposure to CTXs and accepted diagnostic tests for direct detection of CTXs in humans are not available; therefore, diagnosis of ciguatera relies on clinical observations of its overt symptoms and/or testing remnants of consumed fish, if available. Several methods have been developed to screen for the presence of CTXs in fish tissue prior to consumption. In vivo whole-animal detection methods are now superseded by in vitro assays that have greater sensitivity, including receptor-binding assays (RBAs), cell-based assays (CBAs), Enzyme-Linked Immunosorbent Assays (ELISA), capillary electrophoresis (CE)-based immunoassays, electrochemical immunosensors (ECS), and liquid chromatography tandem mass spectrometry (LC-MS/MS). Currently employed methods are compared in Table 4. Present methods for CTX analysis, in general, are labor-intensive, time-consuming, and require laboratory facilities with well-trained technicians. To date, these methods have not been properly validated. At present it is difficult to obtain sufficient standard CTXs as reference calibrants, impeding corroboration and widespread application of these analytical techniques.

Detection of CTXs in fish tissue is not simple, due to their generally low concentrations, the presence of multiple CTX congeners, the limited amount or lack of CTX reference materials for many derivatives, the difficult synthesis or lack of CTX antibodies, the co-occurrence of interfering molecules in fish tissue, and the typically unpredictable incidence of CTXs. Not all of the currently used methods for quantification of CTXs offer sufficient sensitivity, specificity and selectivity. The mouse bioassay (MBA) and radioligand receptor-binding assays (R-RBA) are expected in the near future to be replaced in laboratories due to their low sensitivity, lack of specificity, and high cost, as well as ethical and safety concerns. The fluorescence-based receptor-binding assay (F-RBA) eliminates safety issues associated with radioactive compounds, and its wider application is expected in the future, especially due to the commercial availability of SeaTox^®^ F-RBA test kit. One of the most successful screening methods seems to be the cell-based assay (CBA) using mouse neuroblastoma cells (N2a). This method is simple and sufficiently sensitive, but is time consuming and reflects only the combined effect of the various CTXs present in fish extract. Further, other toxins can also block sodium channels, therefore, the method fails to provide information on toxin profiles. Immunosensors and immunoassays in the form of ELISA or CE-based tests are sensitive and selective tools for ciguatoxin detection, however, they are limited to the availability of CTX-antibodies. Although, in principle, several toxins can be targeted simultaneously, assays typically contain one or a very limited number of antibodies, therefore these methods do not provide information about toxin profiles. Detecting specific toxins can be misleading because toxin profiles can vary considerably among fish. Interestingly, electrochemical biosensors, except for CE and the recently constructed electrode array ECS, have not been developed for CTX detection in fish tissue, possibly due to the lack of sufficient amounts and types of CTX antibodies. Solving the antibody problem in the future may stimulate large-scale development in this field, especially since antibody-based methods offer the potential for miniaturization and development of portable devices and POC tests [139,140,141]. The application of LC-MS/MS for CTXs detection was an important break-through in CTX screening, because this method is sufficiently sensitive, selective, and unique in providing toxin profiles. The major obstacle of its wide-scale application is the lack of widely available reference toxins for quantification. Currently, state-of-the-art CTX detection involves a combination of CBA-N2a assay and LC-MS/MS, where CBA-N2a assay is used for screening the total toxicity of the sample, and LC-MS/MS to confirm the toxins and for providing toxin profiles [19,45,51,87]. LC-MS/MS together with reference materials can fulfill the role of the sensitive and specific CTX detection method, however, it requires highly trained operator and laboratory facilities. Further the method cannot be miniaturized and field application is not possible.

Testing fish tissue for CTXs is of crucial importance because typically muscle tissue is consumed by humans. Such testing, however, requires a highly invasive sampling method, therefore it is not suitable for testing protected fish species, environmental risk assessment, or clinical diagnosis of human ciguatera fish poisoning. Collecting blood samples is not particularly invasive, and has an added advantage that blood is a much less complicated matrix for analysis than muscle tissue. Unfortunately, few studies have been conducted for testing CTXs in fish blood samples, and the possible relationship between the concentrations of CTXs in muscle tissue and blood, if one indeed exists, is not yet known—elucidation of the functional association between these two parameters though statistical modelling would be highly beneficial towards effective screening of the toxin. It is, however, expected that circulating CTX content in blood is higher after a meal of ciguateric fish. Future research will likely focus in this area because it is especially important for identifying CTXs in humans following its intoxication.

Presently, there is great demand for a portable, fast, reliable, easy-to-use, and cheap CTX screening assay for private customers, fishermen, and fish vendors to test their food or catch before consuming or selling it. These point-of-care tests, however, currently do not exist, notwithstanding that performance requirements may be lower than for those methods used in laboratories. CTX testing is still in the hand of specialists, and specialized laboratories are now able to provide sufficiently accurate information about toxin profile and toxin concentration in fish. Although significant advances have been made to develop and improve the performance of ciguatera assays, the ideal assay, one that is simple, rapid, reliable, robust, highly sensitive, quantitative, provides specific toxin profiles, cheap and does not require trained operators and specialized equipment, does not exist to date; developing one is the key challenge for future research on this field.

## Figures and Tables

**Table 1:** Molecular formula and mass (in Da) of identified ciguatoxins ^1^.

**Pacific CTXs**			**Caribbean CTXs**		
P-CTX-1	C_60_H_86_O_19_	1110.6 [37]	C-CTX-1 ^5^	C_62_H_92_O_19_	1140.6 [35]
52-*epi*-P-CTX-1	C_60_H_86_O_19_	1110.6 [38]	C-CTX-2 ^5^	C_62_H_92_O_19_	1140.6 [35]
54-*epi*-P-CTX-1	C_60_H_86_O_19_	1110.6 [38]	C-CTX-1141a	n.a.	1140.6 [34]
54-*epi*-52-*epi*-P-CTX-1	C_60_H_86_O_19_	1110.6 [38]	C-CTX-1141b	n.a.	1140.6 [34]
P-CTX-2 ^2^	C_60_H_86_O_18_	1094.6 [33]	C-CTX-1141c	n.a.	1140.6 [34]
P-CTX-3 ^2^	C_60_H_86_O_18_	1094.6 [33]	C-CTX-1127	n.a.	1126.6 [34]
7-oxo-P-CTX-1	C_60_H_86_O_20_	1126.6 [38]	C-CTX-1143	n.a.	1142.6 [34]
6,7-diH-7-OH-P-CTX-1	C_60_H_88_O_20_	1128.6 [38]	C-CTX-1143a	n.a.	1142.6 [34]
3,4-diH-4-OH-7-oxo-P-CTX-1	C_60_H_88_O_21_	1144.6 [38]	C-CTX-1157	n.a.	1156.6 [34]
54-deoxy-50-OH-P-CTX-1	C_60_H_86_O_19_	1110.6 [38]	C-CTX-1157a	n.a.	1156.6 [34]
P-CTX-3C ^3^	C_57_H_82_O_16_	1022.6 [39]	C-CTX-1157b	n.a.	1156.6 [34]
P-CTX-3B ^3^	C_57_H_82_O_16_	1022.6 [38]	C-CTX-1159	n.a.	1158.6 [34]
51-OH-P-CTX-3C	C_57_H_82_O_17_	1038.6 [40]			
2,3-diH-2-OH-P-CTX-3C	C_57_H_84_O_17_	1040.6 [38]	**Indian CTXs**		
2,3-diH-2,3-diOH-P-CTX-3C	C_57_H_84_O_18_	1056.6 [40]	I-CTX-1	C_62_H_92_O_19_	1140.6 [15]
M-*seco*-P-CTX-3C	C_57_H_84_O_17_	1040.6 [41]	I-CTX-2	C_62_H_92_O_19_	1140.6 [15]
P-CTX-4A ^4^	C_60_H_84_O_16_	1060.6 [42]	I-CTX-3	C_62_H_92_O_20_	1156.6 [15]
P-CTX-4B ^4^	C_60_H_84_O_16_	1060.6 [37]	I-CTX-4	C_62_H_92_O_20_	1156.6 [15]
M-*seco*-P-CTX-4A/B	C_60_H_86_O_17_	1078.6 [41]	I-CTX-5	C_62_H_90_O_19_	1138.6 [19]
51-OH-2-oxo-CTX-3C	C_57_H_82_O_18_	1054.6 [38]	I-CTX-6	C_62_H_90_O_20_	1154.6 [19]
2,3-diH-2,3,51-triOH-P-CTX3C	C_57_H_84_O_19_	1072.6 [38]			
A-*seco*-2,3-diH-51-OH-P-CTX-3C	C_57_H_86_O_18_	1058.6 [38]			

^1^ OH = hydroxy, H = hydro, n.a. = not available; ^2^ Epimers; ^3^ Epimers; ^4^ Epimers; ^5^ Epimers. Alternative or old names: P-CTX-1 = CTX-1B and CTX; P-CTX-2 = 52-*epi*-54-deoxy-CTX-1B; P-CTX-3 = 54-deoxy-CTX-1B; P-CTX-3B = 49-*epi*-P-CTX-3C; P-CTX-4B = 52-*epi*-P-CTX-4A, GTX-4B, GT-4B or gambiertoxin-4B; 49-*epi*-P-CTX-3C = P-CTX-3B; 56-*epi*-C-CTX-1 = C-CTX-2; 2,3-dihydro-2,3-dihydroxy-P-CTX-3C = 2,3-dihydroxy-P-CTX-3C = CTX-2A1.

**Table 3:** Examples of the application of LC-MS for CTX detection.

Method	Fish Species (Family)	Toxin Detected	Mass ^1^ (g)	Conc. ^2^ (ng g^−1^)	LOQ ^3^ (ng g^−1^)	Ref.
LC-MS/MS	*Lutjanus bohar* (Lutjanidae)	P-CTX-1 P-CTX-2 P-CTX-3	5	3.7 0.74 0.36	0.32	[134]
LC-MS/MS	*Sphyraena putnamae* (Sphyraenidae)	P-CTX-1	2	11.4	0.07	[53]
LC-MS/MS	*Sphyraena putnamae* (Sphyraenidae)	P-CTX-1 P-CTX-2 P-CTX-3	2	5.6 7.9 1.4	n.a.	[20]
LC-MS/MS	*Epinephelus spilotoceps* (Serranidae)	P-CTX-1	5	2.73	0.01	[48]
LC-MS/MS	*Variola louti* (Serranidae)	P-CTX-1 P-CTX-2 P-CTX-3	5	2.0 ^4^	<0.01	[41]
LC-MS/MS	*Cephalopholis argus* (Serranidae)	P-CTX-1 P-CTX-2 P-CTX-3	5	1.710 0.555 0.711	0.0005 0.0050 0.0050	[87]
LC-MS/MS	*Gymnothorax flavimarginatus* (Muraenidae)	P-CTX-1 P-CTX-2 P-CTX-3	5	39.20 24.40 5.940	0.0005 0.0050 0.0050	[87]
LC-MS/MS	*Scomberomorus commerson* (Scombridae)	P-CTX-1	5	0.13	n.a.	[135]
LC-HRMS	*Variola louti* (Serranidae)	P-CTX-1	10	1.609	~0.4	[67]
LC-MS/MS	*Pagrus Pagrus* (Sparidae)	C-CTX-1	15	0.76	0.0045	[36]
LC-MS/MS	*Lutjanus cyanopterus* (Lutjanidae)	C-CTX-1	15	0.49	0.0045	[36]
LC-MS/MS	*Seriola fasciata* (Carangidae)	C-CTX-1	15	0.84	n.a.	[51]
LC-MS/MS	*Mycteroperca fusca* (Serranidae)	C-CTX-1	15	0.25	0.0150	[136]
LC-MS/MS	*Caranx lugubris* (Carangidae)	C-CTX-1	100	13.79	n.a.	[137]
LC-HRMS	*Bodianus scrofa* (Wrasse)	C-CTX-1	15	n.a.	n.a.	[138]
LC-HRMS	*Carcharhinus leucas* (Carcharhinidae)	I-CTX-1,2 I-CTX-3,4	10 ^5^	6.54 9.74	1.67 ^6^	[19]

^1^ Mass of fish tissue used for the analysis; ^2^ Concentration of toxin (ng toxin/g fish tissue); n.a. = not available; ^3^ Due to the relationship between the limit of detection, LOD (S/N > 3), and limit of quantitation, LOQ (S/N > 10), only LOQ is shown in table (ng toxin/g fish tissue); ^4^ total toxicity; ^5^ Shark stomach; ^6^ in P-CTX-1 equivalent.

**Table 4:** Comparison of methods for CTX detection.

Method	Extract Preparation ^1^	Assay Duration	Parallel Samples ^2^	Sensitivity ^3^ (ng g^‒1^)	Specificity	Toxin Profile
MBA	5–6 h	24 h	1–10	0.56	No	No
R-RBA	5–6 h	3–4 h	96	0.03–0.15	No	No
F-RBA	5–6 h	2–3 h	96	0.02–0.023	No	No
CBA	5–6 h ^4^	53 h ^4^	96	0.001–0.13	No	No
ELISA	7–8 h	2–4 h	96	<0.01 ^5^	Yes	No
CE	5–6 h	1 h	1	<0.01 ^5^	Yes	No
ECS	5–6 h	2 h	1	0.01	Yes	No
LC-MS/MS	5–8 h	5–15 min ^6^	1	0.0005–0.32	Yes	Yes

^1^ Depends on purity requirements. Parallel samples can be prepared simultaneously depending on lab conditions and operator. Extraction time is different depending on the protocol used. See also Figure 3. As an example, the estimated time for preparing the raw extract is 5–6 h, and for the two SPE purification step is 2 h; ^2^ 96-well microtiter plate is typically used for RBA, CBA and ELISA; however, sample throughput can be 96–1436; ^3^ LOQ for P-CTX-1 equivalent toxin in fish tissue (protocol dependent), note that the suggested tolerance limit for P-CTX-1 in fish tissue is 0.01 ng g^−1^, respectively; ^4^ Includes 24-h incubation and 24-h exposure of the neuro-2a cells to fish extracts, and 4–6 h assay time; extract preparation can be undertaken during incubation time; ^5^ LOD, no specific measurement for LOQ was done; ^6^ This estimation considers only the time of a single injection of a sample into the LC-MS system, and does not includes calibration and quality controls.
